# MULTIPLE SOURCES OF SOCIAL STATUS IN OLD AGE: THE ROLE OF AGE GROUPS AND GENERATIONS

**DOI:** 10.1093/geroni/igz038.2671

**Published:** 2019-11-08

**Authors:** David Weiss, Xin Zhang

**Affiliations:** 1 Leipzig University, Leipzig, Germany; 2 Peking University, Beijing, Beijing, China (People’s Republic)

## Abstract

This cross-cultural study compared attitudes towards age and generational groups across the life span in China, Germany, and the US including N = 1302 participants between 18 and 86 years of age. We asked younger, middle-aged, and older respondents to rate either age (e.g., adolescents, young adults, middle-aged adults, and older, adults) or generational groups (e.g., Millennials, Generation X, Baby Boomer, and Silent Generation) on various characteristics. Results demonstrate that across all three cultures older age groups were perceived consistently less positive and more negative, whereas older generations were perceived as significantly more positive and less negative. Our results suggest that generations in contrast to age groups represent a source of high social status in later life providing a sense of respect, value, and admiration. Thus, social status can be derived from multiple sources and older adults can draw upon alternative social status domains (their generation) when confronted with loss.

